# Evaluation of a self-administered iPad^®^-based processing speed assessment for people with multiple sclerosis in a clinical routine setting

**DOI:** 10.1007/s00415-024-12274-8

**Published:** 2024-03-05

**Authors:** Stefanie Hechenberger, Birgit Helmlinger, Christian Tinauer, Emanuel Jauk, Stefan Ropele, Bettina Heschl, Sebastian Wurth, Anna Damulina, Sebastian Eppinger, Rina Demjaha, Michael Khalil, Christian Enzinger, Daniela Pinter

**Affiliations:** 1 Research Unit for Neuronal Plasticity and Repair, Medical University of Graz, Graz, Austria; 2 Department of Neurology, Medical University of Graz, Graz, Austria; 3 Department of Medical Psychology, Psychosomatics, and Psychotherapy, Medical University of Graz, Graz, Austria; 4 Clinical Psychology and Behavioral Neuroscience, Technische Universität Dresden, Dresden, Germany; 5 Division of Neuroradiology and Interventional Radiology, Department of Radiology, Medical University of Graz, Graz, Austria; 6 Neurology Biomarker Research Unit, Medical University of Graz, Graz, Austria; 7 Head of Research Unit for Neuronal Plasticity and Repair, Department of Neurology, Medical University of Graz, Auenbruggerplatz 22, 8036 Graz, Austria

**Keywords:** Multiple sclerosis, Cognition, Processing speed, Cognitive assessment, MRI, iPad^®^-based test

## Abstract

**Background:**

Limited resources often hinder regular cognitive assessment of people with multiple sclerosis (pwMS) in standard clinical care. A self-administered iPad®-based cognitive screening-tool (Processing Speed Test; PST) might mitigate this problem.

**Objective:**

To evaluate the PST in clinical routine.

**Methods:**

We investigated the feasibility of the PST in both a quiet and a waiting room setting. We assessed the validity of the PST in comparison with the established Symbol Digit Modalities Test (SDMT). We explored associations between processing speed assessments and the Brief International Cognitive Assessment for MS (BICAMS), magnetic resonance imaging (MRI) parameters, and psychological factors. Additionally, we explored the ability of the PST to detect impairment in processing speed compared to the SDMT.

**Results:**

The PST was feasible in the waiting room setting. PST and SDMT correlated comparably with the BICAMS, MRI parameters, and psychological variables. Of 172 pwMS, 50 (30.8%) showed cognitive impairment according to the BICAMS; respective values were 47 (27.3%) for the SDMT and 9 (5.2%) for the PST.

**Conclusions:**

The PST performed in a waiting room setting correlates strongly with established cognitive tests. It thus may be used to assess processing speed in a resource-efficient manner and complement cognitive assessment in clinical routine. Despite comparable validity of the PST and SDMT, we identified more pwMS with impaired processing speed using normative data of the SDMT compared to the PST and advise caution, that the common cut-off score of – 1.5 SD from the current PST is not appropriate in Europe.

**Supplementary Information:**

The online version contains supplementary material available at 10.1007/s00415-024-12274-8.

## Introduction

People with multiple sclerosis (pwMS) are often challenged with so-called invisible symptoms such as cognitive impairment (CI) [1]. Cognitive dysfunction ranges from 34 to 65% over all phenotypes in MS [2] and has a significant effect on patients’ workableness and their quality of life [3]. Deficits in processing speed represent the most common difficulty [2], and have been hypothesized as the key deficit underlying higher-order cognitive dysfunction in domains such as memory and executive function [4].

Over the past three decades, various neuropsychological test batteries have been developed. Several studies support the use of the Symbol Digit Modalities Test (SDMT) to assess processing speed in MS [5, 6]. In addition to its easy and brief administration, the SDMT has clinical and scientific advantages [5]. For instance, it has a high test–retest reliability and good validity, alternative forms with consistent reliability exist, it is able to discriminate between pwMS and healthy individuals, and correlates with neurological symptoms and MS-related brain damage over time [5, 7–9]. Consequently, the SDMT has become part of the most widely used MS-specific neuropsychological test batteries, such as the Brief Repeatable Battery for Neuropsychological Test (BRB), and the Brief International Cognitive Assessment in MS (BICAMS; currently recommended for use as cognitive screening-tool in pwMS by an expert consensus committee) [5, 10].

Due to the high demands and lack of trained personnel, cognitive assessment in clinical practice is quite challenging [11]. Importantly in this context, recent studies have shown the great potential of testing cognitive function in MS using computer-based tools [12–14]. Computer-based cognitive assessment has several advantages over traditional paper–pencil tests, such as standardized administration, automated scoring, and real-time feedback. Furthermore, it can be easily adapted for use in remote settings (e.g., telemedicine appointments) [12, 13]. Therefore, a self-administered computer-based screening-tool could assist clinicians in the routine assessment of cognitive function.

In this study, our *first aim* was to evaluate the feasibility of a self-administered iPad^®^-based screening-tool (Processing Speed Test; PST) [13, 15] to assess processing speed in a clinical waiting room environment. Our *second aim* was to examine the validity of the PST. For this, we explored the association of PST scores with the commonly used paper–pencil SDMT. Furthermore, we examined associations between the processing speed assessments and the currently recommended cognitive screening (BICAMS), MS-related MRI brain tissue changes [2], as well as potential influencing factors, such as depression, anxiety, and fatigue in pwMS [16]. Lastly, our *third aim* was to explore the ability of the PST to detect impairment in processing speed compared to the well-established SDMT.

## Methods

### Participants

A total of 172 pwMS (105 females [61%]; mean age 39.2 ± 9.6 years), diagnosed with a clinically isolated syndrome or definite MS (relapsing–remitting MS, secondary progressive MS, primary progressive MS) underwent clinical, neuropsychological, and brain MRI assessments at the University Hospital of Graz, Austria. All participant data were assessed between October 2020 and May 2022. The time interval between neuropsychological and MRI assessment was not more than eight weeks.

Exclusion criteria for pwMS were an acute relapse/steroid treatment within eight weeks prior to the neuropsychological and MRI assessments and/or other relevant neurological/psychiatric diseases.

To further evaluate PST-performance in our specific setting, we examined 49 healthy controls (HC; 34 females, 69%; mean age 33 ± 11 years), applying the same procedures. HC had to have no chronic neurologic/psychiatric or other relevant diseases/medication.

This project was approved by the Ethics Committee of the Medical University of Graz (31–432 ex 18/19 1264-2019). Written informed consent was obtained from all participants. The study was performed in accordance with the Declaration of Helsinki.

### Neuropsychological assessment

Neuropsychological assessment included three paper–pencil tests (SDMT; Verbal Learning and Memory Test, VLMT; Brief Visuospatial Memory Test, BVMT), the iPad®-based processing speed test (PST), two psychological questionnaires, and one short questionnaire to evaluate participants’ satisfaction with the self-administered iPad®-assessment.

#### Processing speed test

**Fig. 1:**
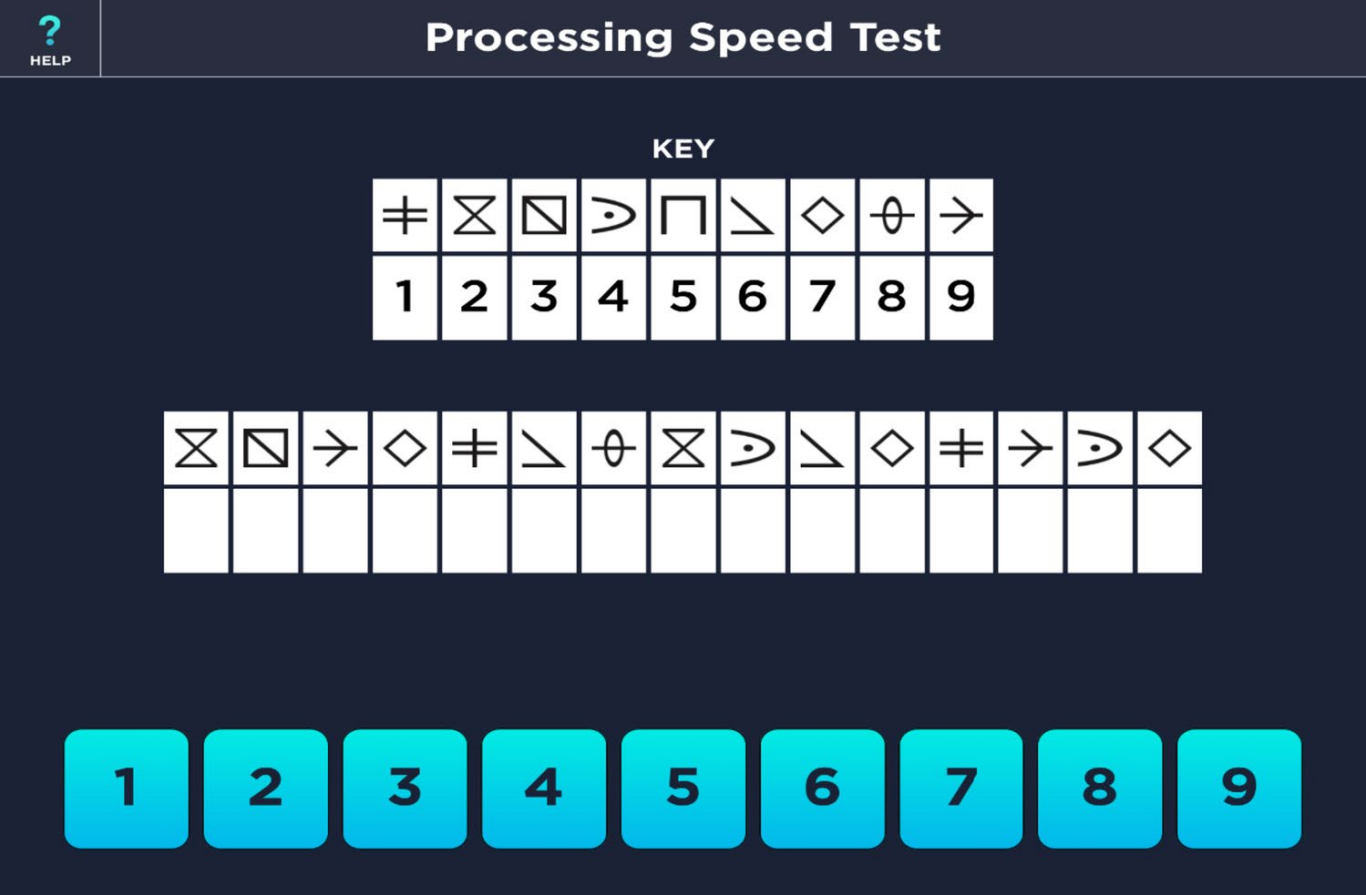
Screenshot of the self-administered, iPad^®^-based cognitive screening-tool: the PST. Screenshot of the PST (App: CogEval by Biogen, 2022) [15]: The PST is a self-administered, iPad^®^-based cognitive screening-tool to assess processing speed in pwMS. PwMS see nine different symbols with a different number. After a detailed instruction and a short training session, pwMS had 2 min time to insert the right number below each symbol (white box in the middle) following a specific key. *PST* Processing Speed Test, *pwMS* people with multiple sclerosis

Processing speed was assessed with a self-administered, iPad^®^-based screening-instrument (PST; Fig. 1) [13, 15] both in a quiet and a waiting room setting. After a detailed instruction and a short training session, participants had two minutes time to insert the correct number below each symbol following a specific key. *Z*-scores corrected for sex, age, and education were automatically generated after the assessment using normative data evaluated in 428 healthy, cognitively intact adults in the United States (US) [17].

#### Brief International Cognitive Assessment for MS

For cognitive screening, we applied the German version of the BICAMS [10, 18], including three subtests for processing speed (SDMT), verbal learning and memory (VLMT), and visuospatial learning and memory (BVMT). Raw scores of the SDMT [19], VLMT [20], and BVMT [21] were transformed to normative scores (*z*-scores) correcting for demographics (SDMT: age, education [19]; VLMT: age [20]; BVMT: age [21]) using respective norms of each cognitive assessment. CI in one cognitive test was defined by a *z*-score < -1.5 [7].

#### Psychological questionnaires

Levels of depression and anxiety were assessed with the Hospital Anxiety and Depression Scale (HADS) [22]. Fatigue was assessed with the Fatigue Scale for Motor and Cognitive Function (FSMC) [23].

### Administration of the neuropsychological assessment for pwMS and HC

The neuropsychological examination started with the BICAMS (SDMT, VLMT, BVMT), followed by the questionnaires (HADS, FSMC), and ended with the PST, which took place twice to test for differences between the quiet and waiting room setting at a within-subjects level. The assessment was performed, either first in the quiet (90 pwMS/25 HC) or in the waiting room (82 pwMS/24 HC) setting. The order for the first PST setting was randomized for all participants (pwMS & HC). In the quiet setting, a psychologist was present in the room, but was not allowed to give instructions, answer questions, or provide feedback to the participants. Due to COVID-19 restrictions, it was not possible to conduct the PST in the original waiting room of our department. Thus a waiting room situation was simulated. Participants were asked to sit in a corridor of our department to perform the PST. During the assessment, participants were exposed to background music from the radio and/or the conversations of passing clinic staff. Participants had a short break (approximately five minutes) after the first PST run. The test setting was then changed and the second PST run was performed.

### Clinical assessment

Specialized neurologists assessed the clinical phenotype, level of physical impairment (Expanded Disability Status Scale; EDSS [24]), and provided information on annual relapse rate, disease modifying therapy (DMT), and disease duration in pwMS.

### MRI protocol

MRI of the brain was performed on a 3T scanner (Siemens MAGNETOM 3T Prisma Fit system) at the Department of Radiology, Medical University of Graz, Austria. To enable assessment of normalized cortical/subcortical brain volumes, high resolution 3D images were acquired using a T1-weighted MPRAGE sequence with 1mm isotropic resolution (repetition time (TR) 1900ms, echo time (TE) 2.7ms). A T2-weighted 3D Fluid-Attenuated Inversion Recovery (FLAIR) sequence with 1mm isotropic resolution was used for the assessment of hyperintense T2 white matter lesion-load (T2-LL) in pwMS (TR 5000ms, TE 393ms, inversion time (TI) 1800ms).

All images were examined by clinicians with expertise in neuroradiology.

### Structural MRI analyses

To assess T2-LL, hyperintense white matter lesions were segmented with the Lesion Segmentation Toolbox on SPM 12, using the automated lesion prediction algorithm (LPA) [25] on FLAIR images. Afterwards, a binary lesion mask (threshold = 0.25) was generated with fslmaths (FSL, v5.9) and the T2-LL (volume in mm^3^) of each patient was extracted using fslstats (FSL) [26].

After lesion-filling with the FSL lesion-filling toolbox [27], brain volumes were assessed based on T1-weighted MPRAGE images using SIENAX [28], part of the FMRIB Software Library (FSL) [26]. All brain volumes were normalized for head size using the V-scaling factor derived by SIENAX. Subcortical volumes (thalamus/hippocampus) were determined from T1-weighted images using FSL FIRST [29]. The volumes estimation pipeline is freely available online (https://github.com/neuroimaging-mug/ms-volest).

### Statistical analyses

All data were analyzed with the Statistical Package of Social Science (IBM SPSS Statistics 29). The level of significance was set to *p* < 0.05. Shapiro–Wilk test was applied to assess normal distribution of all variables and we controlled for outliers. Outliers (more than 3.0 times the interquartile range above or below the quartile) were excluded from the entire analyses. Since four pwMS had a T2-LL that would have met the criteria for an outlier, we performed a log-transformation for this variable. Using this method, we did not have to exclude any participant from the entire analyses.

The false discovery rate (FDR) adjustment of p-values for multiple comparison correction was used where necessary.

Correlations (Pearson/Spearman) were computed to explore associations between the different cognitive tests, and of the cognitive tests with psychological and MRI data. An ANOVA (mixed design with *test-setting* as within-subject factor and *sequence of the test setting* as between-subject factor) was carried out to test for differences in the PST-performance (*z*-scores) between the quiet vs. waiting room setting and to investigate potential sequence or practice effects. ANOVAS (between-subject design) or *t*-tests were performed to explore differences between pwMS and HC in demographics, neuropsychological, and MRI data. We checked assumptions for the correlations and the ANOVAS.

Given the difference in cognitive function between progressive/atypical and relapse-remitting MS, we performed sensitivity analysis excluding pwMS with progressive/atypical phenotypes (*N* = 16).

## Results

### Patients characteristics

Detailed information on demographics, clinical, neuropsychological, and MRI parameters of pwMS/HC are presented in Table 1.

**Table 1:** Patients’ characteristics

Demographics and clinical data	PwMS N = 172	HC N = 49	*p*
Age, mean (SD) (years)	39.2 (9.6)	33.5 (10.7)	< 0.001*
Sex (female), *N* (%)	105 (61)	34 (69)	0.322
Education, mean (SD) (years)	14.2 (3.6)	17 (3.3)	< 0.001*
Disease duration, mean (SD) (years)	10.3 (7.6)	N.A	
EDSS, median (IQR)	1.0 (2.5)	N.A	
Annual relapse rate, mean (SD)	0.5 (0.5)	N.A	
DMT, *N* (%)	135 (78.5)	N.A	
Clinical phenotype, *N* (%)			
CIS	23 (13.4)	N.A	
RRMS	133 (77.3)	N.A	
SPMS	11 (6.4)	N.A	
PPMS	4 (2.3)	N.A	
Atypical	1 (0.6)	N.A	
Cognitive tests			
PST, quiet room, mean (SD)	0.22 (0.96)	0.49 (0.88)	0.043*
PST, waiting room, mean (SD)	0.24 (0.97)	0.55 (0.77)	0.033*
SDMT, mean (SD)	– 0.64 (1.16)	– 0.14 (0.90)	0.005*
VLMT, mean (SD)	0.75 (0.85)	1.12 (0.75)	0.008*
BVMT, mean (SD)	0.56 (1.54)	0.56 (0.86)	0.852
BICAMS, total score, mean (SD)	0.38 (0.82)	0.62 (0.59)	0.026*
Psychological factors			
Fatigue, mean (SD)	48.7 (19.3)	36.5 (11)	< 0.001*
HADS–A, mean (SD)	5.6 (3.9)	3.9 (2.6)	< 0.001*
HADS–D, mean (SD)	3.5 (3.4)	1.7 (1.7)	< 0.001*
Brain MRI parameters			
T2-LL, median (IQR) (cm^3^)	3.1 (5.9)	N.A	
NBV, mean (SD) (cm^3^)	1519 (83.3)	1583 (76.1)	< 0.001*
Thalamus volume, mean (SD) (cm^3^)	15.6 (2.0)	16.6 (1.6)	< 0.001*
Hippocampus volume, mean (SD) (cm^3^)	7.6 (0.9)	7.9 (0.7)	0.010*

*PwMS* people with multiple sclerosis, *HC* healthy controls*,* *N* number of participants; SD: standard deviation, *N.A.* not available, *EDSS* Expanded Disability Status Scale, *IQR* inter quartile range, DMT disease modifying therapy, *CIS* clinically isolated syndrome, *RRMS* relapse remitting multiple sclerosis, *SPMS* secondary progressive multiple sclerosis, *PPMS* primary progressive multiple sclerosis, *PST* Processing Speed Test, *SDMT* symbol digit modalities test, *VLMT* verbal learning and memory test, *BVMT* brief visuospatial memory test, *CI* cognitive impairment, *HADS-A* Hospital Anxiety and Depression Scale-Anxiety; *HADS-D* Hospital Anxiety and Depression Scale-Depression, *MRI* magnetic resonance imaging; *T2-LL* T2-lesion-load, *NBV* normalized brain volume

*PST* *z*-scores based on the normative data from the US, automatically provided from the app [17]

*SDMT* *z*-scores are based on the normative data from Scherer and collegues (2004) [19]

*VLMT* *z*-scores are based on the normative data from Helmstaedter and collegues (2001) [20]

*BVMT* *z*-scores are based on the normative data from Benedict and collegues (1997) [21]

^*^Indicates *p* < 0.05; *N* = 172; *N* = 49 HC

Our pwMS cohort had minor physical disabilities (EDSS median = 1.0, IQR = 2.5).

**Fig. 2:**
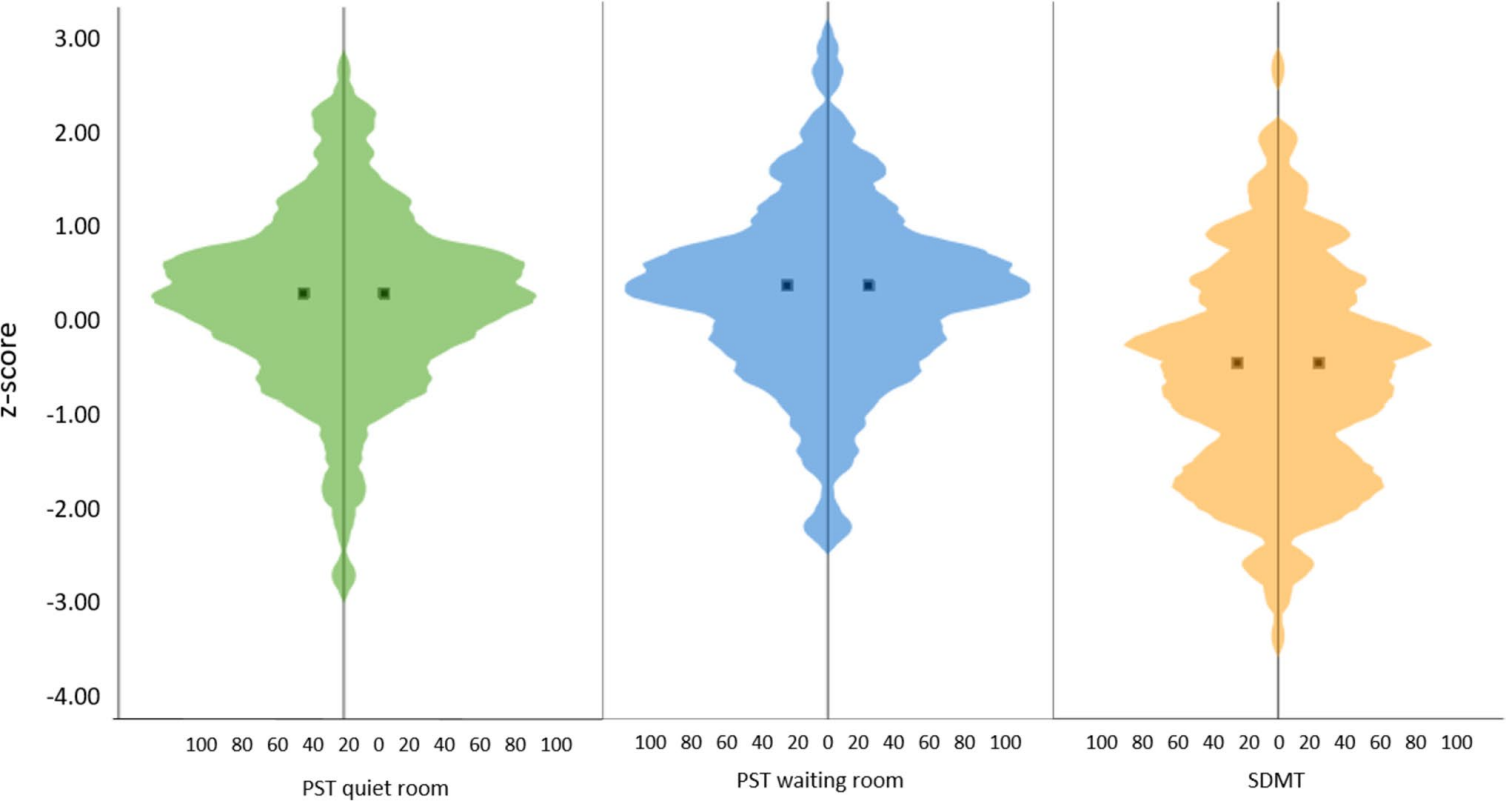
*Z*-score distributions of the PST (quiet and waiting room) and the SDMT. The violin plots show the distribution of *z*-scores (y-axis) and the number of pwMS (x-axis) having a specific *z*-score in the PST (quiet and waiting room setting) and the SDMT. The two dots in the middle of the violin plots represent the mean *z*-score of the cohort in the specific test. *PST* Processing Speed Test, *SDMT* Symbol Digit Modalities Test

All raw values of the neuropsychological tests were standardized using the respective test norms (*z*-score). Regarding processing speed (Fig. 2), pwMS had a mean *z*-score of 0.22 (SD = 0.96, min. = – 2.84, max. = 2.69) in the PST quiet setting, a mean *z*-score of 0.24 (SD = 0.97, min. = – 2.37, max. = 2.97) in the PST waiting room setting and a mean *z*-score of – 0.64 (SD = 1.16, min. = – 3.48, max. = 2.66) in the SDMT.

### Performance and satisfaction with the PST in different test settings

Regarding the administration of the PST, no significant difference in the test performance was found between the quiet (*M* = 0.22, SD = 0.96) and the waiting room (*M* = 0.24, SD = 0.97) setting (*F*_1,170_ = 0.01, *p* = 0.970). However, independent of the test setting, pwMS performed better the second time (*F*_1,170_ = 95.12, *p* < 0.001). More precisely, pwMS starting with the quiet setting (*M* = 0.09, *SD* = 0.99) had a mean *z*-score of 0.43 in the waiting room setting (SD = 0.98, *p* < 0.001). PwMS starting with the waiting room setting at first (*M* = 0.03, SD = 0.93) had a mean *z*-score of 0.37 in the quiet setting (SD = 0.92, *p* < 0.001).

Also, HC performed comparable in the quiet and waiting room setting (Table 1), showing better performance at the second run (*F*_1,47_ = 15.28, *p* < 0.001).

Furthermore, at a descriptive level (5-point rating-scale), patients were highly satisfied with the administration, the given instructions on the iPad^®^-based PST (*M* = 4.67, SD = 0.66) and performance of the neuropsychological screening without any assistance (*M* = 4.47, SD = 0.73).

Satisfaction scores of HC were comparable (administration & instructions:* M* = 4.47, SD = 0.93; performing without assistance: *M* = 4.35, SD = 0.72).

### Validity of the PST

#### Associations of the PST and the SDMT with VLMT, and BVMT

Regardless of the test setting, performance in the PST was highly correlated with the paper–pencil SDMT-performance (raw and *z*-scores).

Furthermore, PST raw and *z*-scores correlated moderately with VLMT, BVMT, and the BICAMS total score. Similar correlations were found between SDMT and VLTM, BVMT, and BICAMS total score (see Table 2). Correlations were also comparable when using the raw values of all cognitive assessments (see Table 2), circumventing possible influence of cross-cultural norms available for the PST (US) and SDMT (Europe).

**Table 2:** Validity of the PST: Correlations between PST / SDMT and BICAMS, MRI parameters, and psychological factors for pwMS

	PST (QR) raw score, *r* (p)	PST (QR) *z*-score, *r* (p)	PST (WR) raw score, *r* (p)	PST (WR) *z*-score, *r* (p)	SDMT raw score, *r* (p)	SDMT *z*-score, *r* (p)
Cognitive tests, *z*-scores						
SDMT, raw score	0.85 (< 0.001*)	0.76 (< 0.001*)	0.84 (< 0.001*)	0.74 (< 0.001*)		
SDMT, *z*-score	0.72 (< 0.001*)	0.78 (< 0.001*)	0.73 (< 0.001*)	0.78 (< 0.001*)	0.92 (< 0.001*)	
VLMT, raw score	0.46 (< 0.001*)	0.36 (< 0.001*)	0.47 (< 0.001*)	0.37 (< 0.001*)	0.48 (< 0.001*)	0.40 (< 0.001*)
VLMT, *z*-score	0.38 (< 0.001*)	0.37 (< 0.001*)	0.42 (< 0.001*)	0.40 (< 0.001*)	0.42 (< 0.001*)	0.40 (< 0.001*)
BVMT, raw score	0.54 (< 0.001*)	0.42 (< 0.001*)	0.50 (< 0.001*)	0.36 (< 0.001*)	0.52 (< 0.001*)	0.41 (< 0.001*)
BVMT, *z*-score	0.49 (< 0.001*)	0.44 (< 0.001*)	0.44 (< 0.001*)	0.38 (< 0.001*)	0.48 (< 0.001*)	0.42 (< 0.001*)
BICAMS, raw score	0.79 (< 0.001*)	0.67 (< 0.001*)	0.78 (< 0.001*)	0.65 (< 0.001*)	0.88 (< 0.001*)	0.78 (< 0.001*)
BICAMS, *z*-score	0.69 (< 0.001*)	0.68 (< 0.001*)	0.68 (< 0.001*)	0.66 (< 0.001*)	0.79 (< 0.001*)	0.78 (< 0.001*)
MRI parameters						
T2-LL	– 0.26 (< 0.001*)	– 0.26 (< 0.001*)	– 0.28 (< 0.001*)	– 0.27 (< 0.001*)	– 0.26 (< 0.001*)	– 0.26 (< 0.001*)
NBV	0.40 (< 0.001*)	0.24 (0.003*)	0.43 (< 0.001*)	0.27 (< 0.001*)	0.39 (< 0.001*)	0.25 (0.001*)
Thalamus vol	0.31 (< 0.001*)	0.32 (< 0.001*)	0.26 (< 0.001*)	0.27 (< 0.001*)	0.22 (0.003*)	0.21 (0.005*)
Hippocampus vol	0.25 (0.001*)	0.26 (< 0.001*)	0.23 (0.003*)	0.23 (0.003*)	0.21 (0.007*)	0.19 (0.011*)
Psychological factors						
Fatigue	– 0.30 (< 0.001*)	– 0.23 (0.007*)	– 0.32 (< 0.001*)	– 0.24 (0.006*)	– 0.26 (< 0.001*)	– 0.17 (0.041*)
Level of depression	– 0.23 (0.007*)	– 0.16 (0.060)	– 0.28 (< 0.001*)	– 0.21 (0.010*)	– 0.22 (< 0.001*)	– 0.16 (0.049*)
Level of anxiety	– 0.10 (0.200)	– 0.12 (0.134)	– 0.14 (0.090)	– 0.17 (0.049*)	– 0.09 (0.247)	– 0.10 (0.200)

*PST* Processing Speed Test, *QR* quiet room setting, *WR* waiting room setting, *SDMT* Symbol Digit Modalities Test, *r* correlation coefficient, *p* *P* value; *VLMT* Verbal Learning and Memory Test, *BVMT* Brief Visuospatial Memory Test, *BICAMS* Brief International Cognitive Assessment for Multiple Sclerosis, *T2-LL* T2 lesion-load, *NBV* normalized brain volume, *vol* volumes

^*^Indicates *p* < 0.05; *N* = 172

*PST* *z*-scores based on the normative data from the US, automatically provided from the app [17]

*SDMT* *z*-scores are based on the normative data from Scherer and collegues (2004) [19]

*VLMT* *z*-scores are based on the normative data from Helmstaedter and collegues (2001) [20]

*BVMT* *z*-scores are based on the normative data from Benedict and collegues (1997) [21]

Associations between processing speed assessments and BICAMS scores for HC are reported in Table S1 (Supplement).

#### Associations with brain MRI parameters

PST and SDMT performance showed similar correlations with brain MRI. Better PST and SDMT performance were significantly associated with lower T2-LL, higher whole brain volume, and higher thalamus and hippocampus volumes (see Table 2). Similarly to the processing speed assessments, VLMT, BVMT, and the BICAMS total score were associated with MRI parameters (Table S2, Supplement).

We found no significant associations between processing speed assessments and MRI parameters in HC (see Table S1, Supplement).

#### Associations with psychological factors

PST *z*-score in the quiet setting was significantly correlated with fatigue (*r* = – 0.23, *p* = 0.007), but not with levels of depression (*r* = – 0.16, *p* = 0.060) or anxiety (*r* = – 0.12, *p* = 0.134). PST *z*-score in the waiting room setting was weakly associated with fatigue (*r* = – 0.24, *p* = 0.006), level of depression (*r* = – 0.21, *p* = 0.010) and level of anxiety (*r* = – 0.17, *p* = 0.049). SDMT *z*-score was significantly associated with fatigue (*r* = – 0.17, *p* = 0.041*) and level of depression (*r* = – 0.16, *p* = 0.049*), but not with level of anxiety (*r* = -0.10, *p* = 0.200). Similarly to the processing speed assessments, VLMT, BVMT, and the BICAMS total score were associated with individual psychological factors (see Table S2, Supplement).

We found no significant associations between processing speed assessments and psychological factors in HC (see Table S1, Supplement).

### Detection of processing speed impairment

In our cohort, 53 (30.8%) pwMS showed CI in at least one cognitive test of the BICAMS. Regarding processing speed, impairment was found in 47 (27.3%) pwMS with the SDMT and in 9 (5.2%) pwMS with the PST (quiet/waiting room setting).

It is noteworthy, that according to the mean PST score of our HC sample (see Table 3) 35 (20.3%) pwMS reached *z*-scores < – 1.5 in processing speed in the quiet and 37 (21.5%) pwMS in the waiting room setting.

**Table 3:** Impairment in processing speed

CI defined by *z*-score < – 1.5	PwMS	HC
CI in PST, quiet room, *N* (%)	9 (5.2)	0 (0)
CI in PST, waiting room, *N* (%)	9 (5.2)	0 (0)
CI in SDMT, Scherer norms, *N* (%)	47 (27.3)	0 (0)
CI in PST, quiet room, according to our HC, *N* (%)	35 (20.3)	2 (4.1)
CI in PST, waiting room, according to out HC, *N* (%)	37 (21.5)	3 (6.1)
CI in SDMT, according to our HC, *N* (%)	46 (26.7)	1 (2)

*CI* cognitive impairment, *PwMS* people with multiple sclerosis, *HC* healthy controls, *PST* processing speed test, *N* number of participants, *SDMT* Symbol Digit Modalities Test

*N* = 172; *N* = 49 HC

*PST* *z*-scores based on the normative data from the US, automatically provided from the app [17]

*SDMT* *z*-scores are based on the normative data from Scherer and collegues (2004) [19]

### Sensitivity analysis focusing on pwMS with CIS and RRMS

In line with the above mentioned results, we found no significant difference in the PST-performance between the quiet (*M* = 0.28, SD = 0.94) and the waiting room (*M* = 0.28, SD = 0.95) setting (*F*_1,154_ = 0.07, *p* = 0.799), if pwMS with progressive and atypical MS phenotypes were excluded (*N* = 16).

The correlations between PST and SDMT with BICAMS, MRI parameters, and psychological factors were almost unaltered (Table S3, Supplement).

In this cohort of pwMS with CIS or RRMS, 46 (29.5%) pwMS showed CI in at least one cognitive test of the BICAMS. Impairment in processing speed was found in 40 (25.6%) pwMS with the SDMT and in 7 (4.5%) pwMS with the PST (quiet/waiting room setting).

## Discussion

Our results provide some support for the application of an iPad^®^-based, self-administered cognitive screening-tool in clinical routine. The PST shows high correlations with the paper–pencil original, the SDMT, and moderate correlations with the two other cognitive tests of the currently recommended standard cognitive assessment (BICAMS). Moreover, individuals were very satisfied with the self-administered assessment on an iPad^®^. Despite comparable validity of the PST and SDMT, we identified more pwMS with impaired processing speed (47 vs. 9) using the normative data of the SDMT [19] compared to the PST [17] and advise caution, that the common cut-off score of -1.5 SD from the current PST is not appropriate in Europe.

PwMS frequently suffer from cognitive dysfunction, particularly in processing speed [2]. In addition, changes in processing speed are known to underlie higher order cognitive dysfunction (e.g., memory, executive functions) [4]. Therefore, the SDMT, which measures processing speed, is currently the recommended processing speed screening-tool for pwMS [2, 16]. Due to factors related to staffing and time, an iPad^®^-based, self-administered screening-tool indeed could assist clinicians in their daily routine [13]. As the iPad^®^-based PST correlates highly with the paper–pencil SDMT, we assume that conducting a self-administered test to measure processing speed on an iPad^®^ is feasible in a clinical setting. Furthermore, similar to the SDMT, the PST showed moderate associations with all subtests (verbal learning and memory, visuospatial memory) of the current standard cognitive screening in MS (BICAMS).

Additionally, pwMS were very satisfied with the instructions, training, and test administration on the PST. Moreover, our results show that the test setting has no significant influence on the performance. Similar scores were obtained in both, the quiet and waiting room setting. The results of our study, therefore, support the application of a self-administered, iPad^®^-based cognitive screening-tool in clinical routine. Since the screening-tool can be used in a waiting room, pwMS could perform the processing speed screening while waiting for their medical appointment.

However, it has to be mentioned that in our cohort, more pwMS were identified as cognitively impaired in processing speed with the SDMT than with the PST, even when compared to our healthy controls, who had the same specific test setting and underwent the same procedure as the pwMS. To generate a standardized *z*-score from the raw scores of the SDMT, we used the normative data of Scherer and colleagues (2004) [19]. These *z*-scores were generated from a cohort of 241 healthy German individuals aged between 19 and 60 years [19], and are almost overlapping those patients performing 1.5 SD below the mean of our HC sample. In comparison, the PST has its own normative data from an American and a Japanese cohort [17, 30]. Data for the American norms were collected from a cohort of 428 healthy, cognitively intact adults from the United States aged between 18 and 89 years [17]. Using the US normative data in an American cohort of pwMS, a recent study has shown that the PST is a sensitive test to detect impairment in processing speed [14]. The Japanese sample included 254 healthy volunteers aged between 20 and 65 years [30]. Interestingly, it was found that the Japanese cohort had higher mean PST scores than the compared US sample, leading to a potential underestimation of impairment in processing speed in the Japanese sample when applying the US normative data. It is, therefore, suggested to use culturally appropriate normative data to gain more accurate normative PST scores [30]. Furthermore, another study generally reported discrepancies in normative data for cognitive tests between American and European nations [31]. Due to differences in test-taking attitudes and education-related factors, normative data may vary between America and Austria [31]. Therefore, clinicians should keep in mind that cross-cultural normative data may affect a patient's outcome. Consequently, in a future study, it would be of great interest to evaluate normative data of the PST in a European sample.

Another finding of our study showed that, regardless of the test environment, pwMS performed better in the PST in the second condition (quiet or waiting room setting). When performing the test twice, it seems that pwMS become familiar with the iPad^®^-test and this may show practice effects [32]. Learning/practice effects are not a unique phenomenon for the PST and were also reported in other studies with the SDMT [32–34]. Nevertheless, it should be mentioned that neither the PST nor the SDMT were developed for repeated use within one neuropsychological assessment, which was specific to our study design. To avoid potential learning effects, cognitive assessment should, therefore, be performed at longer time intervals [33].

MRI volumetric measures, in particular of the thalami and hippocampi, strongly correlate with cognitive changes in pwMS [35, 36]. Moreover, cortical lesions were robustly associated with cognitive decline [37]. In our cohort, we only found moderate correlations between PST/SDMT and total lesion-load, and thalamic and hippocampal volumes. Additionally, correlation coefficients of PST and SDMT with MRI measures were comparable. These results underline the good criterion validity of the PST [38].

Another point to note is that cognitive function and performance is not completely independent of psychological factors such as depression, anxiety, and fatigue [39, 40]. Higher levels of depression and anxiety are associated with worse cognitive function and perceived cognitive complains [39, 40]. The role of fatigue in relation to CI is not yet fully understood. However, a weak impact of fatigue on cognitive performance has been reported [23]. Our results show that PST-performance in both settings was associated with subjectively perceived fatigue. Furthermore, PST-performance in the waiting room was correlated with levels of depression and anxiety. Since we found comparable results of the processing speed assessments, these highlight the validity of the PST. However, our results show that cognitive performance is not sufficiently independent of affective psychological factors. Accordingly, a detailed medical-hostor-taking of influencing factors such as depression, anxiety, and fatigue is important for a cognitive screening [16, 23].

Given all these considerations, it is crucial to highlight that a neuropsychological assessment that includes tests for multiple cognitive domains (e.g., BICAMS) and questionnaires for psychological factors (e.g., mood, fatigue) is superior to the resource efficient sole assessment of processing speed and should be preferred whenever possible [10, 16].

This study is not without limitations. Due to the COVID-19 pandemic, it was not possible to perform the PST in the real waiting room of our department. However, we simulated a waiting room situation with patients performing the PST in a corridor with background noises. Secondly, due to the novelty of the PST, European/German normative data were not available and, hence, we used the available US norms. Lastly, it should also be mentioned that our HC had a significantly higher level of education, which should not have a strong impact on the results due to the use of standardised norms.

In conclusion, a self-administered processing speed assessment in pwMS might be a screening option if (neuro)psychological resources are not available or scarce. PwMS were very satisfied with the PST and it also correlated strongly with the paper-based SDMT. Moreover, PST was also associated with MS-related MRI parameters and psychological factors. However, despite comparable validity of the PST and SDMT, we identified more pwMS with impaired processing speed using the normative data of the SDMT compared to the PST. Therefore, we advise caution: the common cut-off score -1.5 SD from the current PST is not appropriate in Europe, and whenever possible, a comprehensive neuropsychological assessment should be preferred and considered the “gold-standard”, especially for a refined and comprehensive baseline assessment.

### Supplementary Information

Below is the link to the electronic supplementary material.

Supplementary file1 (DOCX 21 KB)

Supplementary file2 (DOCX 19 KB)

Supplementary file3 (DOCX 21 KB)

## Data Availability

The datasets generated during and/or analyzed during the current study are not publicly available due to the use of sensitive patient data but are available from the corresponding author on reasonable request.
