# Supplementary material for: Evaluation of a self-administered iPad®-based processing speed assessment for people with multiple sclerosis in a clinical routine setting
Source: J Neurol. 2024 Mar 5;271(6):3268–78. doi: 10.1007/s00415-024-12274-8 (PMC11136781; doi:10.1007/s00415-024-12274-8)
Supplement: Supplementary file 1 — Supplementary file1 (DOCX 21 KB) [file 415_2024_12274_MOESM1_ESM.docx]

**Evaluation of a Self-administered iPad^®^-based Processing Speed Assessment for People with Multiple Sclerosis in a Clinical Routine Setting**

Stefanie Hechenberger^1,2^, Birgit Helmlinger^1,2^, Christian Tinauer^2^, Emanuel Jauk^3,4^, Stefan Ropele^2^, Bettina Heschl^2^, Sebastian Wurth^2,5^, Anna Damulina^2^, Sebastian Eppinger^2,5^, Rina Demjaha^2,6^, Michael Khalil^2,6^, Christian Enzinger^1,2^, Daniela Pinter^1,2^

^1^ Medical University of Graz, Research Unit for Neuronal Plasticity and Repair, Graz, Austria

^2^ Medical University of Graz, Department of Neurology, Graz, Austria

^3^ Medical University of Graz, Department of Medical Psychology, Psychosomatics, and Psychotherapy, Graz, Austria

^4^ Technische Universität Dresden, Clinical Psychology and Behavioral Neuroscience, Dresden, Germany

^5^ Medical University of Graz, Division of Neuroradiology & Interventional Radiology, Department of Radiology, Graz, Austria

^6^ Medical University of Graz, Neurology Biomarker Research Unit, Graz, Austria

***Corresponding Author:**

Daniela Pinter, PhD

Department of Neurology, Head of Research Unit for Neuronal Plasticity and Repair, Medical University of Graz; Auenbruggerplatz 22, 8036 Graz, Austria

Email: daniela.pinter@medunigraz.at

Phone: 0043 316 385 31215

**Table S1. Correlations between PST / SDMT and BICAMS, MRI parameters, and psychological factors for HC.**

|  | **PST (QR)  raw score, *r* (p)** | **PST (QR)   z-score, *r* (p)** | **PST (WR) raw score,  *r* (p)** | **PST (WR)  z-score, *r* (p)** | **SDMT raw score,  *r* (p)** | **SDMT  z-score, *r* (p)** |
| --- | --- | --- | --- | --- | --- | --- |
| **Cognitive tests,**  **z-scores** |  |  |  |  |  |  |
| SDMT, raw score | 0.73 (<0.001*) | 0.54 (<0.001*) | 0.72 (<0.001*) | 0.59 (<0.001*) |  |  |
| SDMT, z-score | 0.60 (<0.001*) | 0.61 (<0.001*) | 0.53 (<0.001*) | 0.60 (<0.001*) | 0.87 (<0.001*) |  |
| VLMT, raw score | 0.27 (0.109) | 0.18 (0.422) | 0.32 (0.052) | 0.27 (0.170) | 0.41 (0.008*) | 0.33 (0.076) |
| VLMT, z-score | 0.17 (0.386) | 0.18 (0.422) | 0.21 (0.259) | 0.26 (0.170) | 0.24 (0.188) | 0.27 (0.177) |
| BVMT, raw score | 0.37 (0.025*) | -0.02 (0.903) | 0.48 (<0.001*) | 0.11 (0.736) | 0.44 (0.007*) | 0.19 (0.358) |
| BVMT, z-score | 0.28 (0.109*) | 0.04 (0.903) | 0.36 (0.028*) | 0.13 (0.644) | 0.40 (0.010) | 0.23 (0.257) |
| BICAMS, raw score | 0.62 (<0.001*) | 0.37 (0.035*) | 0.66 (<0.001*) | 0.46 (<0.001*) | 0.85 (<0.001*) | 0.67 (<0.001*) |
| BICAMS, z-score | 0.49 (<0.001*) | 0.39 (0.023*) | 0.50 (<0.001*) | 0.46 (<0.001*) | 0.73 (<0.001*) | 0.68 (<0.001*) |
| **MRI parameters** |  |  |  |  |  |  |
| T2-LL | N.A. | N.A. | N.A. | N.A. | N.A. | N.A. |
| NBV | 0.13 (0.512) | -0.11 (0.631) | 0.12 (0.545) | -0.15 (0.636) | 0.17 (0.360) | 0.05 (0.877) |
| Thalamus vol. | 0.03 (0.841) | 0.02 (0.903) | 0.02 (0.951) | -0.04 (0.843) | 0.05 (0.816) | 0.08 (0.877) |
| Hippocampus vol. | -0.07 (0.784) | 0.04 (0.903) | -0.12 (0.545) | -0.02 (0.843) | -0.09 (0.645) | -0.05 (0.877) |
| **Psychological factors** |  |  |  |  |  |  |
| Fatigue | -0.04 (0.841) | 0.16 (0.481) | -0.15 (0.459) | 0.04 (0.843) | -0.12 (0.520) | -0.02 (0.984) |
| Level of depression | 0.05 (0.841) | 0.11 (0.631) | -0.06 (0.719) | -0.01 (0.927) | 0.02 (0.892) | -0.01 (0.984) |
| Level of anxiety | 0.27 (0.109) | 0.24 (0.272) | 0.10 (0.581) | 0.05 (0.843) | 0.19 (0.317) | 0.18 (0.358) |

HC: healthy controls; PST: processing speed test; QR: quiet room setting: WR: waiting room setting; SDMT: Symbol Digit Modalities Test; r: correlation coefficient; p: P-value; VLMT: Verbal Learning and Memory Test; BVMT: Brief Visuospatial Memory Test; BICAMS: Brief International Cognitive Assessment for Multiple Sclerosis; T2-LL: T2 lesion load; NBV: normalized brain volume; vol: volumes

* indicates p<0.05; N=49

PST: z-scores based on the normative data from the US, automatically provided from the app [17]

SDMT: z-scores are based on the normative data from Scherer and collegues (2004) [19]

VLMT: z-scores are based on the normative data from Helmstaedter and collegues (2001) [20]

BVMT: z-scores are based on the normative data from Benedict and collegues (1997) [21]
