# Supplementary material for: Evaluation of a self-administered iPad®-based processing speed assessment for people with multiple sclerosis in a clinical routine setting
Source: J Neurol. 2024 Mar 5;271(6):3268–78. doi: 10.1007/s00415-024-12274-8 (PMC11136781; doi:10.1007/s00415-024-12274-8)
Supplement: Supplementary file 2 — Supplementary file2 (DOCX 19 KB) [file 415_2024_12274_MOESM2_ESM.docx]

**Evaluation of a Self-administered iPad^®^-based Processing Speed Assessment for People with Multiple Sclerosis in a Clinical Routine Setting**

Stefanie Hechenberger^1,2^, Birgit Helmlinger^1,2^, Christian Tinauer^2^, Emanuel Jauk^3,4^, Stefan Ropele^2^, Bettina Heschl^2^, Sebastian Wurth^2,5^, Anna Damulina^2^, Sebastian Eppinger^2,5^, Rina Demjaha^2,6^, Michael Khalil^2,6^, Christian Enzinger^1,2^, Daniela Pinter^1,2^

^1^ Medical University of Graz, Research Unit for Neuronal Plasticity and Repair, Graz, Austria

^2^ Medical University of Graz, Department of Neurology, Graz, Austria

^3^ Medical University of Graz, Department of Medical Psychology, Psychosomatics, and Psychotherapy, Graz, Austria

^4^ Technische Universität Dresden, Clinical Psychology and Behavioral Neuroscience, Dresden, Germany

^5^ Medical University of Graz, Division of Neuroradiology & Interventional Radiology, Department of Radiology, Graz, Austria

^6^ Medical University of Graz, Neurology Biomarker Research Unit, Graz, Austria

***Corresponding Author:**

Daniela Pinter, PhD

Department of Neurology, Head of Research Unit for Neuronal Plasticity and Repair, Medical University of Graz; Auenbruggerplatz 22, 8036 Graz, Austria

Email: daniela.pinter@medunigraz.at

Phone: 0043 316 385 31215

**Table S2. Correlations between VLMT, BVMT, BICAMS total score and MRI parameters and psychological factors in pwMS**

|  | **VLMT  raw score, *r* (p)** | **VLMT   z-score, *r* (p)** | **BVMT raw score,  *r* (p)** | **BVMT   z-score, *r* (p)** | **BICAMS raw score,  *r* (p)** | **BICAMS  z-score, *r* (p)** |
| --- | --- | --- | --- | --- | --- | --- |
| **MRI parameters** |  |  |  |  |  |  |
| T2-LL | -0.20 (<0.001*) | -0.13 (0.094) | -0.29 (<0.001*) | -0.27 (<0.001*) | -0.29 (<0.001*) | -0.29 (<0.001*) |
| NBV | 0.35 (<0.001*) | 0.24 (0.002*) | 0.34 (<0.001*) | 0.25 (<0.001*) | 0.44 (<0.001*) | 0.32 (0.002*) |
| Thalamus vol. | 0.16 (0.046*) | 0.13 (0.093) | 0.28 (<0.001*) | 0.28 (<0.001*) | 0.26 (<0.001*) | 0.27 (0.007*) |
| Hippocampus vol. | 0.07 (0.351) | 0.05 (0.543) | 0.23 (0.003*) | 0.24 (0.003*) | 0.21 (0.009*) | 0.21 (0.014*) |
| **Psychological factors** |  |  |  |  |  |  |
| Fatigue | -0.18 (0.038*) | -0.13 (0.098) | -0.22 (0.014*) | -0.24 (0.012*) | -0.27 (0.002*) | -0.19 (0.038*) |
| Level of depression | -0.15 (0.085) | -0.14 (0.097) | -0.19 (0.038*) | -0.18 (0.038*) | -0.23 (0.012*) | -0.18 (0.038*) |
| Level of anxiety | -0.13 (0.106) | -0.16 (0.076) | -0.10 (0.189) | -0.15 (0.086) | -0.13 (0.106) | -0.13 (0.098) |

VLMT: Verbal learning and memory test; BVMT: Brief Visuospatial Memory Test; BICAMS: Brief International Cognitive Assessment for Multiple Sclerosis; MRI: magnetic resonance imaging; T2-LL: T2 lesion load; NBV: normalized brain volume; vol.: volume

* indicates p<0.05; N=172

SDMT: z-scores are based on the normative data from Scherer and collegues (2004) [19]

VLMT: z-scores are based on the normative data from Helmstaedter and collegues (2001) [20]

BVMT: z-scores are based on the normative data from Benedict and collegues (1997) [21]
