# Supplementary material for: Evaluation of a self-administered iPad®-based processing speed assessment for people with multiple sclerosis in a clinical routine setting
Source: J Neurol. 2024 Mar 5;271(6):3268–78. doi: 10.1007/s00415-024-12274-8 (PMC11136781; doi:10.1007/s00415-024-12274-8)
Supplement: Supplementary file 3 — Supplementary file3 (DOCX 21 KB) [file 415_2024_12274_MOESM3_ESM.docx]

**Evaluation of a Self-administered iPad^®^-based Processing Speed Assessment for People with Multiple Sclerosis in a Clinical Routine Setting**

Stefanie Hechenberger^1,2^, Birgit Helmlinger^1,2^, Christian Tinauer^2^, Emanuel Jauk^3,4^, Stefan Ropele^2^, Bettina Heschl^2^, Sebastian Wurth^2,5^, Anna Damulina^2^, Sebastian Eppinger^2,5^, Rina Demjaha^2,6^, Michael Khalil^2,6^, Christian Enzinger^1,2^, Daniela Pinter^1,2^

^1^ Medical University of Graz, Research Unit for Neuronal Plasticity and Repair, Graz, Austria

^2^ Medical University of Graz, Department of Neurology, Graz, Austria

^3^ Medical University of Graz, Department of Medical Psychology, Psychosomatics, and Psychotherapy, Graz, Austria

^4^ Technische Universität Dresden, Clinical Psychology and Behavioral Neuroscience, Dresden, Germany

^5^ Medical University of Graz, Division of Neuroradiology & Interventional Radiology, Department of Radiology, Graz, Austria

^6^ Medical University of Graz, Neurology Biomarker Research Unit, Graz, Austria

***Corresponding Author:**

Daniela Pinter, PhD

Department of Neurology, Head of Research Unit for Neuronal Plasticity and Repair, Medical University of Graz; Auenbruggerplatz 22, 8036 Graz, Austria

Email: daniela.pinter@medunigraz.at

Phone: 0043 316 385 31215

**Table S3. Sensitivity analysis focusing in pwMS with CIS and RMS**

|  | **PST (QR)  raw score, *r* (p)** | **PST (QR)   z-score, *r* (p)** | **PST (WR) raw score,  *r* (p)** | **PST (WR)  z-score, *r* (p)** | **SDMT raw score,  *r* (p)** | **SDMT  z-score, *r* (p)** |
| --- | --- | --- | --- | --- | --- | --- |
| **Cognitive tests,**  **z-scores** |  |  |  |  |  |  |
| SDMT, raw score | 0.83 (<0.001*) | 0.75 (<0.001*) | 0.83 (<0.001*) | 0.73 (<0.001*) |  |  |
| SDMT, z-score | 0.70 (<0.001*) | 0.76 (<0.001*) | 0.72 (<0.001*) | 0.77 (<0.001*) | 0.93 (<0.001*) |  |
| VLMT, raw score | 0.41 (<0.001*) | 0.34 (<0.001*) | 0.42 (<0.001*) | 0.35 (<0.001*) | 0.45 (<0.001*) | 0.38 (<0.001*) |
| VLMT, z-score | 0.34 (<0.001*) | 0.34 (<0.001*) | 0.37 (<0.001*) | 0.38 (<0.001*) | 0.39 (<0.001*) | 0.38 (<0.001*) |
| BVMT, raw score | 0.55 (<0.001*) | 0.43 (<0.001*) | 0.51 (<0.001*) | 0.39 (<0.001*) | 0.56 (<0.001*) | 0.42 (<0.001*) |
| BVMT, z-score | 0.50 (<0.001*) | 0.45 (<0.001*) | 0.46 (<0.001*) | 0.40 (<0.001*) | 0.49 (<0.001*) | 0.43 (<0.001*) |
| BICAMS, raw score | 0.78 (<0.001*) | 0.67 (<0.001*) | 0.77 (<0.001*) | 0.65 (<0.001*) | 0.88 (<0.001*) | 0.78 (<0.001*) |
| BICAMS, z-score | 0.68 (<0.001*) | 0.68 (<0.001*) | 0.67 (<0.001*) | 0.66 (<0.001*) | 0.79 (<0.001*) | 0.78 (<0.001*) |
| **MRI parameters** |  |  |  |  |  |  |
| T2-LL | -0.27 (<0.001*) | -0.26 (0.002*) | -0.27 (<0.001*) | -0.26 (<0.001*) | -0.27 (<0.001*) | -0.26 (0.002*) |
| NBV | 0.36 (<0.001*) | 0.21 (0.009*) | 0.40 (<0.001*) | 0.25 (0.002*) | 0.35 (<0.001*) | 0.22 (0.008*) |
| Thalamus vol. | 0.31 (<0.001*) | 0.32 (<0.001*) | 0.26 (0.002*) | 0.26 (0.002*) | 0.23 (0.007*) | 0.20 (0.013*) |
| Hippocampus vol. | 0.22 (0.007*) | 0.23 (0.006*) | 0.20 (0.014*) | 0.20 (0.014*) | 0.19 (0.019*) | 0.17 (0.031*) |
| **Psychological factors** |  |  |  |  |  |  |
| Fatigue | -0.28 (<0.001*) | -0.18 (0.048*) | -0.30 (<0.001*) | -0.20 (0.027*) | -0.24 (0.014*) | -0.14 (0.124) |
| Level of depression | -0.21 (0.027*) | -0.12 (0.166) | -0.28 (<0.001*) | -0.20 (0.027*) | -0.21 (0.027*) | -0.14 (0.112) |
| Level of anxiety | -0.12 (0.166) | -0.12 (0.173) | -0.17 (0.062) | -0.17 (0.062) | -0.11 (0.199) | -0.10 (0.201) |

**In line with findings of the entire cohort results remained unaltered, except for the correlation between PST (WR, z-score) and level of anxiety, SDMT (z-score) and fatigue, and SDMT (z-score) and level of depression.**

PST: processing speed test; QR: quiet room setting: WR: waiting room setting; SDMT: Symbol Digit Modalities Test; r: correlation coefficient; p: P-value; VLMT: Verbal Learning and Memory Test; BVMT: Brief Visuospatial Memory Test; BICAMS: Brief International Cognitive Assessment for Multiple Sclerosis; T2-LL: T2 lesion load; NBV: normalized brain volume; vol: volumes

* indicates p<0.05; N=156

PST: z-scores based on the normative data from the US, automatically provided from the app [17]

SDMT: z-scores are based on the normative data from Scherer and collegues (2004) [19]

VLMT: z-scores are based on the normative data from Helmstaedter and collegues (2001) [20]

BVMT: z-scores are based on the normative data from Benedict and collegues (1997) [21]
